# Sterno-Mastoid Tumour

**Published:** 1891-07-11

**Authors:** 


### SURGERY.

### STERNO-MASTOID TUMOUR.

This affection, which is known under various names, is of much interest and no small obscurity. It is almost if not quite confined to children, and appears within a few days—at latest a few weeks—after birth, and hence is often called congenital sterno-mastoid tumour.

It is always unilateral, intimately connected with the muscle, and may either involve its whole extent, which then has a board-like hardness, or only a portion of it, imparting a knotty feel. The skin over it is not involved, and is normal, and it is often painful on pressure, or when the affected muscle is put in action. It is accompanied by more or less shortening of the muscle, which is sometimes so marked as to produce a spurious torticollis. Its mobility varies with the state of contraction or relaxation of the sterno-mastoid, in the former case being fixed, and in the latter only moveable from side to side. This feature is important as forming the best diagnostic mark between it and enlarged cervical glands, with which it is apt to be confounded. These are freely moveable, vertically or transversely, lie along one edge of the sterno-mastoid, and are not affected by its condition.

The ætiology of this formation is very obscure, and conflicting views are held by different observers, some crediting hereditary syphilis with the chief share in its causation, while others are inclined to consider traumatism as the most usual exciting cause.

Histologically, the induration appears to be a myo-sclerosis, but whether this is the result of a diffuse gummatous infiltration, or a simple inflammatory exudation it is hard to say. Chronic syphilitic induration occurs in the muscles of adults—chiefly in those of the upper extremity—as a tertiary symptom, and is, to all appearances, similar to that we are discussing. Moreover, several cases have been reported where either other syphilitic lesions existed, or where the prompt reaction to anti-syphilitic remedies pointed to such a connection. Berkeley Hill mentions a case of the first kind, and A. E. Barker records three cases, in two of which the hereditary taint certainly existed, and in one this was uncertain. All three cases subsided steadily under the administration of iodide of potassium, but did not react to mercury. In one of the cases, while the tumour was diminishing under the iodide, its administration was stopped, and immediately the swelling became stationary; but on being resumed the process of absorption began again, and was finally successfully completed.

Bryant considers that some cases may be due to syphilis, and so does Agnew; but they as well as many others have seen cases (and these they consider to be the great majority) where there is no reason to suspect any hereditary taint.

A large section of the profession are inclined to attribute these cases, in the majority of instances, to an injury inflicted during labour, either from an unfavourable presentation, or from the application of forceps.

Dr. Rushton Parker has just published a note of two cases of difficult labour, in which a tumour developed in the sterno-mastoid three or four weeks after birth, and most writers on the subject mention this as a possible cause.

Those who adopt this view explain the occurence as being produced by the rupture of some fibres either from a strain of the muscle during the delivery of the head in some abnormal position—as in breech presentations for instance, or from the direct pressure of the blade of the forceps. More or less blood is entravasated under the sheath of the muscle, and a limited simple inflammation is set up to repair the damage, thus producing a more or less extensive induration. Against this view must, however, be placed the fact that such tumours are seen in cases where the birth has been quite normal and easy, and here we must fall back upon the hypothesis of some muscular injury having occurred in some other way which we cannot recognise or be content to leave it unexplained. It seems to us possible that these different views may be partially harmonised if we suppose that an injury is inflicted on the muscle which would be too trivial to produce any effect in the case of a healthy child, but able where the constitution is weak either from hereditary syphilitic taint or from the strumous diathesis to give rise to a chronic interstitial growth; and this view of the case is borne out by the fact that this condition is generally found in feeble and emaciated children. The prognosis is very good; the affection in some cases subsiding spontaneously. Specific treatment or general tonics, such as cod-liver oil and the syrup of the iodide of iron, may, however, be required in accordance with the facts stated above, while locally a little simple friction over the swelling is recommended.

